# An Opportunity to Be Heard: Family Experiences of Coronial Investigations Into Missing People and Views on Best Practice

**DOI:** 10.3389/fpsyg.2019.02322

**Published:** 2019-11-12

**Authors:** Stephanie Dartnall, Jane Goodman-Delahunty, Judith Gullifer

**Affiliations:** School of Psychology, Charles Sturt University, Bathurst, NSW, Australia

**Keywords:** missing person, families of missing people, inquest, thematic analysis, ambiguous loss, Coroner’s Court, therapeutic jurisprudence, semi-structured interviews

## Abstract

Experiences of 15 family members and friends of missing people of a coronial investigation into the suspected death of a missing person in New South Wales (NSW), Australia were examined via in-depth interviews. This study explored participant perceptions of the impact of coronial proceedings on well-being, and views on best practice approaches to families in the Coroner’s Court. Transcripts were thematically analysed, yielding six key themes in participant experiences of inquests: (1) Opportunity to be heard, (2) A chance for education, (3) If you are human with me (sensitive treatment and language), (4) Timely investigations, (5) A public and formal court environment, and (6) Coronial outcomes. Overall, families benefitted from opportunities to have input and feel heard, compassionate treatment, and appropriate education about the process and available support services. A detriment on well-being was described when these factors were precluded. Some participants perceived positive outcomes arising from public awareness of cases of missing people, formalities that conveyed respect, and timeframes that enabled further investigation or preparation for the inquest. Others reported distress and trauma in response to significant delays that led to a loss of evidence, intrusive media and unknown persons in court, and unwelcoming, formal court environments. Some participants were profoundly distressed by a finding of death and by the procedures that followed the inquest, emphasising the need for post-inquest debriefing and ongoing support. These findings deepen our understanding of coronial practices, and of measures to prevent harm, that will be instructive to other coronial jurisdictions. Further research should examine family experiences in contexts where there are variable coronial proceedings or procedures that result in legal findings of death.

## Introduction

Australian coroners are judicial officers and lawyers who investigate and make findings about reportable deaths and suspected deaths (Dillon, 2015). Coroners seek to establish the facts surrounding a death: if, how, when, where, and why a death occurred (Freckelton and Ranson, 2006; Dillon and Hadley, 2015). Coroners do not determine issues of guilt or liability, nor are they bound by the rules of evidence (Selby, 1998; King, 2008). There is no uniform approach to missing persons’ cases in Australian Coroners’ Courts. Each state and territory differs in its coronial legislation and practices to investigate “suspected deaths” when the person remains unlocated. A study of 322 closed coronial investigations into missing people (suspected deaths) in New South Wales (NSW) revealed that coroners investigated an average of 24 suspected deaths per annum, with the majority resulting in an inquest (96%) and a finding that the missing person was deceased (94%), and few resulting in findings as to manner of death. A total of 18% (58 cases) resulted in a finding of self-inflicted death, homicide, or misadventure. Timeframes of coronial investigations were variable, with over half (52%) of the 322 investigations finalised more than 10 years after the person’s disappearance (Dartnall and Goodman-Delahunty, 2016).

Once a NSW coroner returns a finding of death, it is recorded with the NSW Registry of Births Deaths and Marriages and senior next of kin can apply for a death certificate. In some cases, a death certificate is required to manage the missing person’s estate (NSW Department of Justice Producer, 2017). Internationally, coronial jurisdiction in cases of missing people might only arise if certain conditions are met. Examples are the United Kingdom and New Zealand (Bain, 2011; Coronial Services of New Zealand, 2016; Thornton, 2016; UK Missing Persons Unit, n.d.). The utility of coronial investigations and inquests into missing people is an issue of some contention. As investigators in an inquisitorial legal system, coroners review police evidence to make findings about a suspected death. Coroners also have the capacity to issue search orders, order further police investigation, hold public hearings in a court (inquests), and make recommendations in relation to systemic issues and matters of public health and safety connected to the suspected death (Dillon and Hadley, 2015). In inquests into missing people, NSW coroners formally recommended: rewards for information, referrals to unsolved homicide, and measures to improve rock fishing safety and police investigation procedures (Dartnall and Goodman-Delahunty, 2016). Some advocates contend that mandatory public inquests provide additional benefit in their capacity to: (a) act as a “safeguard” to ensure thorough police investigations; (b) aid in the identification of suspicious cases; (c) attract media and public attention to assist the police investigation; and (d) provide a “therapeutic benefit” for families of missing people (Law Reform Commission of Western Australia, 2012, p. 84; Legal Aid New South Wales, 2016, p. 6).

Whether the inquest produces a therapeutic benefit for the families of missing people, and family experiences of the court process, are issues warranting empirical exploration. Two Australian doctoral research projects qualitatively explored the lived experiences and perceptions of hope of relatives of missing people (Glassock, 2011; Wayland, 2015). Brief comments on coronial investigations contained therein alluded to significant and variable impacts of coronial proceedings on families, and the importance of further research on this topic. Results from one study revealed that for some relatives, a finding of death “quashed” nearly all hope that the missing person would be located alive, while another in the family continued to hope the missing person was “ok” and endeavoured to ignore the coronial findings (Wayland, 2015, p. 241). Glassock (2011) characterised a presumption of death as both “confronting” and “traumatising” for one father who described the significant physical, emotional, and relational toll of the inquest:

As the date of the inquest approaches there is a build-up of tension with potential consequent effects on sleep and general well-being and perhaps on relationships…nothing prepares you for the eventual pronouncement that your son is deceased. When it comes it’s a sledgehammer blow despite the fact that it was anticipated. (Glassock, 2011, p. 151)

The complex and varied nature of family responses to legal declarations of death was evident in the aftermath of 9/11, when relatives in New York were offered “certificates of presumed death” for people whose bodies remained unrecovered. Some relatives found this useful to manage financial affairs, or emotionally helpful, while others declined these certificates, preferring to await proof of death or to accept the presence of ambiguity (Boss, 2002b, p. 16; Boss, 2004).

Internationally, missing persons’ research typically urges understanding that the loss when someone is missing, often termed “ambiguous loss,” differs vastly from bereavement following a death (Boss, 2006). Researchers suggest that pressuring families to accept a particular outcome, or to close the door on a missing loved one, can alienate and distress families, disregard important ongoing connections with missing people, and prevent relatives from moving forward therapeutically (Boss, 2002a; Clark, 2007; Glassock, 2011; Boss and Carnes, 2012). While the missing persons’ research moves beyond traditional concepts of closure and grief work, these concepts abound in the coronial literature (Biddle, 2003). The lens through which we perceive the impact of inquests is largely derived from a handful of studies of cohorts where death is certain, and scholarly articles that posit the potential for coronial investigations to assist families with healing in their potential to offer closure (Biddle, 2003; Freckelton, 2007; King, 2008; Federation of Community Legal Centres Victoria, 2013). Researchers report that families usually expect some measure of closure from coronial investigations and that distress results when definitive answers and closure are delayed or fail to eventuate (Wertheimer, 2001; Davis et al., 2002; Biddle, 2003; Hands, 2012).

Despite urging by scholars and coronial professionals for coronial practice that minimises harm to court participants, surprisingly few published studies have examined family experiences of inquests in Australia (Freckelton, 2006; Freckelton and Ranson, 2006). Family views of coronial proceedings were explored as a component of reviews of Victorian and Western Australian legislation, producing fairly consistent insights into problems encountered in these courts (Victorian Parliament Law Reform Committee, 2006; Law Reform Commission of Western Australia, 2011). A prominent concern in both jurisdictions was a lack of timely and appropriate information resulting in a poor understanding of coronial processes and family rights. In Victoria, examples were cited where next of kin were unaware of their rights to request evidence or to have legal representation, and four next of kin were unable to attend an inquest because they were not advised of the time of the inquest. Delays in coronial proceedings were a significant source of distress for families, particularly due to attrition in evidence, financial strain, and prolonged grieving for families recounting information many years after a death. Disappointment and distress were expressed about other aspects of the coronial process, including insensitive or adversarial courtroom behaviour, poor access to legal representation, and recommendations that were unenforceable or that failed to address systemic issues (Victorian Parliament Law Reform Committee, 2006; Law Reform Commission of Western Australia, 2011).

More recently, Australian in-depth interviews explored family experiences of institutional responses, and family satisfaction with the account of a death, following workplace fatalities. These studies revealed that families were concerned and frustrated by: infrequent updates, a poor understanding of their rights and whether an inquest would be held, and delays that prolonged stress and impaired witness memory (Matthews et al., 2012, 2017; Ngo, 2016). Families valued inquests, and perceived a sense of justice or enhanced trust in the outcomes, when: (a) provided direct access to previously inaccessible evidence, (b) treated with greater respect than in other investigations, (c) permitted to raise opinions or questions in the inquest directly or through legal representation, or (d) the inquest revealed previously unidentified systemic failings that contributed to the death. Families were disappointed, did not value, or felt a sense of injustice, in relation to inquests in cases in which nothing further was revealed, where key witnesses were not called or questioned thoroughly, or where families felt unable to challenge issues, including cases in which families were legally unrepresented (Matthews et al., 2012, 2017; Ngo, 2016; Ngo et al., 2018). Researchers suggested that a key pathway to enhance therapeutic outcomes for families was to provide opportunities during inquests for families to question witnesses and to voice their views on the circumstances of the death (Ngo et al., 2018).

Internationally, a series of studies, most related to suicide inquests, revealed the detrimental impact on families of: (a) poor information about the coronial process that created trepidation and unrealistic, unrealised expectations; (b) delays that left families “in limbo,” diminished evidence, and delayed other outcomes such as the release of suicide notes; (c) intrusive, sensationalist and inaccurate media; and (d) exposure to evidence that was unexpected, graphic, repetitious, inconsistent, dehumanising, or that failed to clarify circumstances surrounding the death or to take into account the family’s version of events (Wertheimer, 2001; Davis et al., 2002; Harwood et al., 2002; Biddle, 2003; Snell and Tombs, 2011; Spillane et al., 2019). In some instances, insensitive, adversarial behaviours and courtroom formalities exacerbated feelings of guilt and criminalisation for families who provided witness testimony (Barraclough and Shepherd, 1976; Wertheimer, 2001; Davis et al., 2002; Biddle, 2003; Snell and Tombs, 2011). Researchers observed significant variation in family responses to findings. For example, some families perceived that undetermined (open) suicide verdicts created ambiguity which exacerbated distress; others preferred open verdicts; and some felt suicide verdicts were appropriate (Wertheimer, 2001; Biddle, 2003; Chapple et al., 2012; Spillane et al., 2019). Examples of positive coronial experiences included cases where families appreciated less formal court settings, opportunities to review the evidence or to attend the court prior to the inquest, face-to-face meetings with court officers, and empathetic coronial professionals expressing genuine condolences (Wertheimer, 2001; Davis et al., 2002; Biddle, 2003).

Therapeutic jurisprudence (TJ) is an approach that encourages research to explore how legal rules, procedures and professionals produce neutral, positive or harmful psychological and physical health consequences for participants (Birgden and Ward, 2003). TJ seeks to consider how laws and legal processes might be adapted to minimise negative therapeutic consequences on participants and increase the potential for positive therapeutic outcomes, without jeopardising important justice values, such as due process (Casey and Rottman, 2000; Birgden and Ward, 2003). Concern for participant well-being and the therapeutic implications of coronial proceedings has been a consideration of Coroners’ Courts. When TJ theories were emerging, Waller (1994) advocated that coroners demonstrate concern by taking time to hear family views and offer genuine condolences. Subsequent papers highlighted therapeutic measures implemented by courts, including: (a) counselling services, (b) timely explanations of the coronial process, (c) family statements, (d) sensitive communication including referring to relatives and “the deceased” by name, (e) allowing photos of “the decedent” in court, and (f) conferences with relatives prior to, or instead of, an inquest (Parry et al., 1996; Freckelton and Ranson, 2006; King, 2008; Roper, 2014; Freckelton, 2016). Coroners implement such measures with discretion to ensure they do not compromise the purpose of the inquest or rights of others (Freckelton, 2016; Roper and Holmes, 2016). Research exploring family experiences of “therapeutic” measures remains limited.

The foregoing review revealed significant gaps in the literature, with no known study focusing on family experiences of Coroners’ Courts where a death is yet to be established, and limited exploration of family views of good practice. The present study addressed these omissions and gave families of missing people a voice in the coronial literature. Specifically, the study aimed to explore and describe family: (1) experiences of the coronial process, (2) views of the impact of coronial proceedings on family well-being, particularly factors perceived to have positive or negative consequences, and (3) opinions on best practice approaches to support and inform families throughout coronial proceedings.

## Materials and Methods

### Participants

Approval for this study was granted by the Charles Sturt University Human Research Ethics Committee (2015/274).

Participants were family members, significant others, or friends of a missing person whose suspected death was investigated by a NSW coroner. All participants were over 18 years of age, spoke English, and were willing to discuss their experience of a finalised coronial investigation. Using purposive sampling, participants were recruited through the mailing lists, Facebook page, website, and events of the Families and Friends of Missing Persons Unit (FFMPU), NSW Department of Justice^1^. Participants were invited to contact the first author if interested in the study. This approach was supplemented by snowball sampling. Family members and professionals in the missing persons sector were invited to share information with other families, agencies, and professionals. Key stakeholders shared Facebook posts about this research, including: the Australian Missing Persons Register^2^, Picnic for Missing^3^, and Leave a Light On^4^. Three Facebook posts each reached audiences of over 1030 people.

The final sample comprised 15 participants after one withdrew. Participants’ demographic profiles are presented in Table 1. Participants provided insights into 14 coronial investigations of 13 missing people. Participants’ experiences with respect to the timing and number of inquests are presented in Table 2. All but one participant attended at least one inquest. At interview, 10 participants (67%) reported that the person had been missing for over 10 years. The sample included two friends of missing persons. One friend acted as a representative for the family at the inquest. Hereafter, the term “family” refers to experiences of all participants, inclusive of friends.

**TABLE 1:** Demographic profiles of participants at time of interview.

	**Percent**	***n***
**Gender**		
Female	73	11
Male	27	4
**Age in years**		
65 and over	27	4
46–64	73	11
18–45	0	0
**Relationship to missing person**		
Parent	67	10
Sibling	13	2
Friend/family representative	13	2
Child	7	1
**Residential location**		
NSW rural	53	8
NSW city	33	5
Interstate	13	2
**Employment**		
Employed	60	9
Retired/pensioner	40	6
Unemployed/student/home duties	0	0
**Highest level of education**		
University	60	9
Trade cert/diploma	20	3
School (HSC or year 10 equiv.)	20	3
**Main home language**		
English	93	14
Other	7	1

**TABLE 2:** Timeframes and number of inquests.

	**Percent**	***n***
**Time (disappearance to inquest)**		
0–1 years	0	0
2–4 years	53	8
5–9 years	0	0
10–14 years	20	3
15–19 years	7	1
20–29 years	0	0
≥30 years	20	3
**Time (inquest to interview)**		
0–1 years	40	6
2–4 years	27	4
5–9 years	27	4
≥10 years	7	1
**No. of inquests held into the missing person**		
1	80	12
2	20	3

*Times were calculated based on the dates of: (a) the missing person’s disappearance/last known sighting, (b) the most recent inquest, and (c) the research interview.*

### Materials and Procedure

Interviews were conducted face-to-face by the first author between March 2016 and May 2018, were digitally recorded, transcribed verbatim, and de-identified. Interviews lasted an average of 92 min, ranging in duration from 50–153 min. The interview commenced with an open-ended question that encouraged participants to share their experience of the coronial process in whatever manner they felt appropriate. Participants were then asked questions from a topic guide (Table 3) to explore issues not already discussed, and to seek further understanding of new issues raised by participants (Punch, 1998).

**TABLE 3:** Interview questions and topic guide.

1	Tell me in your own words what the coronial process was like for you, before, during and after the inquest.
2	What impact has the coronial process had on you/family or friends?
3	What happened during the coronial process that was the most helpful/least helpful to your emotional and physical well-being?
4	What did you expect from the coronial investigation/inquest?
5	What, in your opinion, is best practice in supporting and informing families throughout a coronial investigation?
6	Do you feel you had adequate access to information and advice about the coronial process?
7	Do you feel you had adequate access to counselling and support before, during and after the inquest?
8	Can you describe your participation in coronial proceedings?
9	How fair was the coronial process/how satisfied were you with the coronial process, and why?
10	What findings/recommendations were made?
11	What were your impressions of the court building?
12	What were your overall impressions of the language used in inquests?
13	Do you have any suggestions for change to coronial processes?
14	Would you like to add or suggest anything else?

### Analysis

Analysis was led by the first author who adopted a pragmatic approach. Pragmatism values “workability,” methodologies that best fit the research question, action over philosophising, and theories to inform practice, and improve life (Robson and McCartan, 2016). Truth is viewed as fluctuating and provisional, and research conclusions as neither certain nor perfect but something to be continually revised in light of “what works” in the current environment (Johnson and Onwuegbuzie, 2004).

Interviews were thematically analysed in six recursive phases, summarised in Table 4 (Braun and Clarke, 2006, 2012). Thematic analysis systematically analyses and categorises natural language interview responses into prevalent “themes” that represent patterns of meaning, to tell a story of the data as a whole in response to the research questions posed. NVivo 10-12 software supported coding and the development of themes.

**TABLE 4:** Steps of thematic analysis.

**Step**	**Details**
Familiarisation	Repeat listening to audiofiles to facilitate data immersion; repeat reading of transcripts while noting points of interest, coding ideas, reactions to the data, ideas for new interview questions, repetitive or novel issues, and preliminary thematic ideas.
Inductive coding	Systematic reading of transcripts to identify units of text relevant to the research questions, and to assign each chunk of text a label.
Constructing themes	Analysis of the content of each code and relationship between codes, grouping codes into broader themes. e.g., family statement, family witness, and questioning witnesses, were grouped into a theme provisionally labelled “active participation” because these codes reflected the family’s experience of active contribution to the investigation. Eleven preliminary themes were identified in relation to family experiences of the coronial process and best practice.
Naming themes	Named themes and subthemes to convey the underlying meaning of the theme. For example, “active participation” was renamed “opportunity to be heard” to preserve a phrase used repeatedly by participants.
Themes revision	Explored the consistency of data coded within each theme, differentiation between themes, and the relationships between themes. Themes were identified as salient or predominant if they intersected with a number of other themes, if several codes clustered within that theme, or if participants labelled the concept as important or as having a significant impact on well-being.
Establishing trustworthiness	The reliability of findings was established by: (a) allowing participants to check and edit transcripts, (b) reflexive journaling of research steps, (c) development of a codebook to enhance replicability of decisions, (d) discussion and review of analytic steps with co-authors experienced in qualitative research, (e) mapping thematic concepts, and (f) independently comparing raw data (transcripts) with final themes to validate interpretations (Nowell et al., 2017).

## Results

Analysis of participant responses yielded six interrelated themes of family experiences of the coronial process, listed in order of dominance: (1) Opportunity to be heard, (2) A chance for education, (3) If you are human with me, (4) Timely investigations, (5) A public and formal court environment, and (6) Coronial outcomes.

### Opportunity to Be Heard

This overarching theme intersected with all other themes, and mapped the predominant desire of participants to have input, to be “heard” and understood in the coronial process. This theme encompassed three subthemes: (1) Grieving that never stops: participants’ views on their experience of grief; (2) Being heard: the extent to which participants were able to share their views and to be “heard,” and (3) Best practice: recommendations for family input into coronial proceedings.

#### Grieving That Never Stops

Many participants explained that understanding their experience of the coronial process was predicated on understanding their grief. Most perceived a need for court professionals to be cognizant that their grief was “totally different” from grief following a death. Most described their grief as unending “limbo” that could not be “completed,” a “constant living of pain”:

It never goes away…It’s grieving that never stops. That’s the best way to explain it. Because if we found them and we could put them to rest and then you do your normal grieving process. But you don’t, so it’s just ongoing grieving. (Fiona)

Many described feeling that their experiences were poorly understood by others who tried to “solve an unsolvable problem,” pressured them to attain “closure,” and made “ignominious” statements like “Well, you should be over that by now” (John). For some, this lack of understanding caused withdrawal from support mechanisms. Most described a history of distress caused by poorly explained systems and processes that did not fit their experience. Hayley described the detrimental impact of the lack of rituals and public acknowledgement of missing people: “I want somewhere for him to be remembered. We don’t have a grave, we don’t have a headstone, we don’t have anything.”

#### Being Heard

Having input rather than being a “spectator” of proceedings was a crucial factor impacting well-being for most participants. When participants perceived an opportunity to “speak” and were “heard” this was “therapeutic” and “cathartic” and provided a sense of comfort:

It was a comforting feeling and I think a lot of that had to do with the fact that we were given opportunity to speak. It is like that in everything isn’t it? If you feel like you haven’t had a chance to say what you feel you are not happy. (Elaine)

Conversely it was “distressing” for those who were not provided opportunity for input.

Many perceived it paramount to be able to raise issues with the court prior to the inquest. Two participants emphasised the benefit of taking part in a directions hearing where the coroner encouraged them to share their views, and subsequently their suggestions for further investigation were followed up. For John, this process demonstrated a thorough investigation: “It shows that we have left no stones unturned and neither has the coroner.”

Some participants reflected positively on the opportunity to question witnesses in court through counsel assisting the coroner (counsel assisting), or a legal representative. Alex described the advantage of “acting as counsel for the family” which enabled him to question witnesses and challenge issues from the bar table:

Through the investigation and everything else, you’re a bystander. You do what you can for the side. This time, we were involved integrally in the process. We’re doing something ourselves for [the missing person] that was good.

For other participants, a source of frustration was questions that were not asked of witnesses at the inquest. Some felt deprived of the opportunity to question witnesses because they were unaware they could raise questions or uncertain of the scope of the issues that could be raised, leaving questions they “would have asked” after the inquest. For one participant the least helpful aspect of the coronial process to their well-being was the inability to question a person directly in court because they were not called as a witness.

Participants also acknowledged opportunities to voice their opinions through witness testimony and family statements. One third of participants described inquests in which family members gave witness testimony. For one participant, being a witness was “hard” because they felt ill-prepared, having only two weeks to read the police brief of evidence (the brief) prior to the inquest. Another appreciated the opportunity for multiple family witnesses to express divergent views as to what they believed happened to their missing loved one and why.

Family statements appeared to be the key vehicle for families to feel heard. Almost three quarters of participants provided a statement in court, usually after all the evidence had been presented and before findings were made. In their statement, families shared their views on a range of issues, including: (a) the police investigation, (b) the character of the missing person, (c) family relationships, (d) what they believed happened to the missing person, (e) the impact of their disappearance on the family, and (f) suggestions for improvements to systems. The family statement was the most helpful aspect of the coronial process to well-being for three participants. One participant identified their primary purpose in attending the inquest was to be the “voice” of the missing person:

I went up into the witness box…and I just spoke aboutGrahame … [the family] wanted him not to be just Grahame Smith ‘tick the box’, but rather: this is Grahame the person, this is what he meant to his family, this is how his family has felt since he has gone, this was his life, these were his hopes and their hopes for him…I must have spoken for 20 or 30 minutes and I was given plenty of opportunity to say what I needed to say…[The family] said that is all they wanted, that there was someone there to talk for Grahame, and they were very pleased that I had been given such an opportunity and such a hearing. (Chloe)

For others, their statement was an important opportunity to express concerns about system failures to an authority. Where family members were provided time to say whatever they chose to share, this contributed to perceptions that the process was transparent and meaningful, that “nothing was hidden” (John), and that the missing person’s “life was valued” (Elaine). David observed that while it was “harrowing” providing a statement, because “anger” resurfaced, nonetheless, providing a statement was crucial to well-being and contributed to a sense of fairness:

I think it is very, very important, in fact vital for the person’s own well-being, the well-being of the parents that is, and those who are attending on behalf of the family in the court, that they be given the opportunity to make a statement. I think that element of fairness is really important…They need to be given the opportunity to get some things off their chest… particularly if they have got some issues with the way the police investigation was conducted.

Participants reported “distress” when it was unclear before the inquest whether they would be allowed to speak or what they could say. In one case, it was “horrific” to be asked to reword the family statement the night before presenting it in court. For one participant, the “most damaging” factor was that the person of interest was not required to attend court to hear the family statement and the pain the family had endured; this was perceived as demonstrative of a process that “favoured” the rights of the person of interest over that of families.

The distressing impact of exclusion was demonstrated in two cases in which immediate family did not attend an inquest because next of kin were not notified until after the inquests had occurred. In both cases, the inability to attend when the coroner delivered a finding of death triggered considerable “anger,” perceptions of “unfair” treatment, and confusion about the findings. In each case, two inquests were held, and both participants perceived it crucial to be present and to have opportunity for input at each inquest.

#### Best Practice – Opportunity to Be Heard

Many participants suggested that a central component of best practice was to allow families to express their views before and during court, and for professionals to respond to family concerns and needs. Participants suggested that best practice included seeking family views on: (a) the character of the missing person, (b) what happened to the missing person, (c) necessary corrections to errors in the brief, (d) further police investigation, (e) potential witnesses, (f) the timing of the inquest, and (g) potential recommendations. Where family suggestions for the coronial investigation were infeasible, it was perceived important that the reasons be explained to the family. Allowing family statements was a central component of best practice for a number of participants. While some believed there should be no restrictions on family statements, other participants recommended guidelines or better explanations of family statements:

My biggest thing was: What am I supposed to put in it [the family statement]? There’s no guidelines…on what goes into that. So some sort of guidelines, some sort of framework…That would be really helpful. (Hayley)

One participant urged legislative change to allow voluntary coronial investigations, believing families should have input into whether a coronial investigation would proceed.

### A Chance for Education

This dominant theme incorporated participants’ views on the degree to which they were educated in the lead up to, and during, an inquest, divided into four subthemes: (1) Adequate lines of communication: the perceived adequacy of education about the coronial process; (2) Reading the brief: the experience and impact of reading the brief; (3) Support services: experiences of legal and counselling services associated with the court process; and (4) Best practice approaches to educating families about the coronial process.

#### Adequate Lines of Communication

Seven participants identified that they felt “adequately” informed about the coronial process and as a result, felt more “comfortable” and more able to participate in the inquest. Those who felt adequately informed typically described receiving both written and direct communication from court professionals in phone conversations and face-to-face meetings. Some participants valued the provision of plain language resources:

I read that [coronial guide] cover to cover. I passed it round to the kids and this gave us a really good understanding of what the process would look like. So we knew we didn’t have to be worried about anything. This book, the guide I think was probably one of the most singular things that helped us. (Hayley)

Most participants valued pre-inquest opportunities to speak with counsel assisting, to ask questions, clarify information, and learn case specific information. Participants appreciated opportunities to discuss who would be in the court, courtroom conventions, the rationale for the inquest, likely findings, timeframes, and how families could participate:

They [counsel assisting] recapped essentially what was going to happen, how the process would run, how long it might take, what role Mum would play in it. They explained… whether Mum would have an opportunity to get up and speak. (Lauren)

By contrast, eight participants reported “inadequate” access to information, a poor understanding of their rights, that they could have been better informed, or resorted to self-education about court procedures. A lack of education, knowledge of who to contact for advice, and proactive contact from professionals caused “distress,” “pain,” and “anger.” Anne described anguish from a lack of clarity about the process and professional roles: “I just felt lost all the time because I didn’t know who to contact for what. There was no guidance, no structure to what I needed to do. Even coming from police.” Late notifications meant that some participants felt ill-prepared to participate in the inquest: “They said you can ask questions or talk. So that was–and that was told to us–just before we went in, when we turned up…” (Kate).

#### Reading the Brief

Fourteen participants described their experience of reading the brief prior to the inquest. While reading the brief was “distressing” and “challenging,” most perceived the benefits to outweigh the distress. For David, reading the brief minimised surprises at the inquest and helped to prepare a statement for the court:

You already know what is contained in the brief, so your shocks have come earlier, your surprises have come earlier, so in some ways you are more prepared by doing that…I have heard of some people who don’t have access to the brief … Would I have preferred to have had access to the brief? Yes, although it was painful I think yes, because it enabled me to make some statements in my address to the court.

Some participants observed limits to the family’s capacity to prepare for the evidence they would encounter because unexpected evidence and testimony emerged at an inquest. This was the case for Jennifer who described distress when witnesses “lied” and said “horrible things” about the missing person:

You don’t know what that witness is going to say. Like I thought I was all prepared to hear anything and everything, but when I heard that…there’s no way of pre-warning somebody.

Aspects of reading the brief that were perceived as “challenging” and “hurtful” were: (a) errors such as getting the missing person’s name wrong, (b) “dispassionate” and “dehumanising” terminology, (c) distressing accounts of what might have happened to the missing person, and (d) unexpected information including unknown statements, and unknown aspects of the person’s life. Diana described distress from inadequate time to process unexpected information in the brief prior to the inquest:

Not knowing that this other history was there. I felt so guilty [cries] because you feel you should have known…Having that police brief and being able to have these conversations much earlier would have helped us incredibly…So not knowing that, and not having time to even think about those sorts of things was really, really–it was distressing. Having to constantly request a copy of the brief was also very distressing, and not getting it.

Some participants felt fortunate to have accessed the brief and perceived it “helpful” to gain insight into the thoroughness of the police investigation:

There was just so much stuff… it was great. There were things I didn’t realise that they had done. The police had done. Comments made by different people that said they had sighted Josh after he had gone missing…It was all included and it was a bit of an eye opener to get all the information. So that was quite good. (Elaine)

#### Support Services

For some participants, support services provided crucial preparation for the inquest and assisted them to express their views in court. For others, the absence of appropriate support services was a source of distress and was perceived to negatively impact their opportunity to be heard.

One participant had legal representation at the inquest; a Legal Aid lawyer who attended court meetings, was the main point of communication with the court, and asked questions on behalf of family at the inquest. Nonetheless, this participant expressed frustration over having to conduct independent research to find legal assistance and that some questions were not asked at the inquest. Four participants felt that legal representation would have increased awareness of their rights and expression of their views at the inquest; however, two were unaware of their right to legal representation prior to their inquests, and two from the same inquest described the distress experienced when declined Legal Aid representation: “To be told that your [child’s] murder wouldn’t be a public interest case, that hurts” (Jennifer). These participants were unable to afford private representation which they estimated would likely have cost them their house.

Not all participants perceived a need for legal representation; three felt legal representation would have added nothing. A few sought minor legal advice to understand the process. Alex described a two-hour meeting with Legal Aid as the “most helpful” factor in the coronial process, because he learned about the court process and relevant texts: the *Coroners Act 2009* (NSW) and “Waller’s Coronial Law” (Abernethy et al., 2010). Reading these texts helped him understand the relevant case law, which enabled him to act as family counsel.

Similarly, not all participants were aware of available counselling services (the Coronial Information and Support Program^5^ or FFMPU). In some cases the absence of timely referrals to counsellors was a source of concern. Participants who accessed counselling support appreciated that counsellors could answer questions and were “willing to listen.” Two participants described counselling support as one of the “most helpful” aspects of the coronial process, which assisted them to feel “comfortable” having a voice in the coronial process, as described by Meredith:

[Counsellor] had been with me from the start of the journey…I just felt more comfortable because I didn’t have to keep re-explaining myself…Having [counsellor] was just probably the best thing that happened for me, in terms of being able to ask him questions and run things by him and for someone who’s seen all the different variations of things…I just felt much more confident in myself that I’d get through it.

One third of participants reported positive experiences of a counsellor who accompanied them at the inquest. Six participants described the benefits of attending support groups where they met other families who had experienced inquests, which helped them learn from others and feel less isolated. Some participants felt an important component of their pre-inquest preparation was attending information sessions hosted by counselling services where they learned from coroners and asked questions about the process:

One of the [information days] where we got information from the coroner. That was really helpful…I just wasn’t prepared for what was to come. I wasn’t as aware of the legal implications of somebody being missing for a long time. But I found it quite educational, it really opened my eyes. (Elaine)

#### Best Practice – A Chance for Education

As illustrated by John, appropriate pre-inquest communication was perceived crucial to reduce unnecessary distress and facilitate family voice:

There should be a chance for education to help you through the process…for God’s sake let’s be educated before we go in there so we’re not scared, we are not frightened, we are not shocked, we know what’s coming, and we know we can talk.

Most interviewees suggested that families needed “dialogue” with professionals who could explain the court process and discuss family questions and expectations. Participants suggested that early in a coronial investigation, families should be advised of a key contact person to call for advice at any point, and to contact families with updates. Many recommended family conferences with counsel assisting in the lead-up to an inquest and most emphasised the need for timely communication, with a number of participants suggesting that families should not endure lengthy periods between updates. Participants recommended key points at which to contact families prior to an inquest, including prior to receiving the brief to explain the process and forewarn about potentially distressing evidence: “[best practice is] basically to make contact with the people involved, to inform us when the police brief gets there” (Diana). One third of participants recommended mandatory dissemination of the coronial guide prior to an inquest (Families and Friends of Missing Persons Unit, 2017).

Almost three quarters of participants recommended uniform referrals to legal and counselling services, and many felt court professionals, or police, should check that families were aware of support services. David perceived referrals to provide a measure of reassurance to families:

I certainly hope that the counselling services that are operated through both the FFMPU and the coronial court are continued to be offered to people. Now you might not all want to use them but I do think that families need to have some reassurance from people who are familiar with the process and…know how to advise families who are impacted by this.

Some participants suggested proactive calls from counselling services, or pre-inquest conferences involving counsellors, to discuss the emotional impact of coronial proceedings, coping strategies, and ways to prepare for an inquest. Other participants nominated opportunities to attend support groups and information days. One participant recommended separate pre-inquest conferences for young people, attended by counsellors, to help them understand the process. Two recommended that support personnel attend the inquest to explain court proceedings to families.

### If You Are Human With Me

This theme captured participants’ experiences of the language and conduct of professionals in court: their level of compassion, sensitivity, empathy, and how comprehensible they were; with one “best practice” sub-theme. Many participants articulated that humanity and emotion were essential to facilitate family involvement:

If I’m being questioned and you keep that up, just straight without any emotion, well you will get nothing out of me. If you are going to be human with me, I’ll be human with you. (John)

All but two participants spoke positively about the “empathetic” behaviour of coroners. Seven participants described the sensitive treatment from coronial professionals as the “most helpful” aspect of the process, providing examples of coroners who qualified that “it was sad” or “not easy” to deliver such findings; whose tenor was “warm” and body language attentive “I knew she was listening and nodding when I was speaking” (Lauren); and who made eye contact and addressed each family member by name in court. Meredith described the importance of the coroner’s “gentle” conduct of the inquest and acknowledgement that the hearing did not equate to closure:

I’m pretty sure he even said, this is just part of the process and it won’t bring you closure … Which is why I went, oh thank god! Someone who understands his brief and knows that this is by far not closure at all, it’s just another step in the process…I just felt very comforted by the fact that he got it and he wasn’t just processing a piece of paper.

Two other participants found “most helpful” the compassionate behaviour of investigating police. Lauren noted the “sensitive” way counsel assisting spoke to her mother in the foyer before the inquest was the single most helpful factor to her well-being: “He knelt down, he held Mum’s hand and he explained very kindly again the process and what was likely to happen.”

Families appreciated professionals who: (a) appeared unhurried, (b) “really listened,” (c) were attuned to disparate needs of individuals, (d) took time to check family understanding, (e) exhibited “patience” with family questions, (f) used language that was clear and “not overly couched in legalisms,” and (g) sounded genuine and well-prepared. Meredith deemed the sensitive terminology in the delivery of findings helpful even though the findings were unpalatable: “[The coroner said] Dad was more than likely deceased – and I don’t mind those words, I think that’s much nicer than saying he is, I thought that was good terminology.” By contrast, legal terminology in findings which was unexpected and unexplained, was perceived as “devastating” and one of the “least helpful” aspects of the process for David:

When the letter arrived, I am pretty sure it began with ‘I am satisfied’ and went on to pronounce Simon to be deceased. Now, we thought that was a very poor choice of words because no one can be satisfied that a young person has taken his life or met death by misadventure, which are the probabilities… the ‘I am satisfied’ stuff… that gets to me. Certainly there was no knowledge that that terminology was going to be used.

#### Best Practice – If you Are Human With Me

Participants emphasised that best practice communication was non-blaming, “empathic, understanding, sympathetic, attentive and all those things” (David). A number described the importance of using “non-clinical” language and “layman’s terms.” Many observed the need for professionals to ask families “Are you okay?” The importance of sensitive discretion regarding evidence presented in court was appreciated:

They’ve got all these sensitivities around these peoples’ families and their stories …it’s not protecting a family from some of what might come out but it’s guiding that truth without delving into it. And I think that’s part of the best practice thing that I thought was really good. Enough knowledge without digging looking for dirt. (Hayley)

David suggested more sympathetic language than the legal phrase “I am satisfied” the person is deceased, or alternatively, that families be well-prepared and receive explanations for the conventional terminology.

### Timely Investigations

This theme captured family experiences of the timing of the coronial investigation and their assessment of the investigation as timely or untimely, with one “best practice” sub-theme. Vastly different experiences were reported with respect to the timing, and perceptions of timeliness, of coronial investigations. Some participants experienced inquests two to three years after a disappearance; three participants experienced an inquest more than 30 years after the person’s disappearance. Some inquests lasted “10 minutes” or “half an hour,” others lasted “years.” A few participants perceived lengthy delays as “traumatic” and “tormenting,” mostly in suspicious cases, where: they hoped the coronial investigation would trigger a homicide investigation or uncover new leads; where there was a lack of regular feedback about the coronial investigation; and where crucial evidence was “lost,” “forgotten,” or “destroyed.” Anne emphasised the destructive impact of waiting almost two decades for an inquest:

That’s a long time to try and find out the truth of anything. A lot of information had been lost, a lot had been forgotten, a lot of witnesses couldn’t recall anything… It was like we were searching definitely for a needle in a haystack …There were witnesses that hadn’t been interviewed for years that had very crucial information.

In some historical investigations, entire missing persons’ files were “lost” prior to a report to the coroner, requiring time to collect new statements, exacerbating “trauma”:

For Mum having to retell the story again, you know she had told it so many times …The whole process was made more traumatic than it should have been because of the loss of James’ file. You have to be confident in the knowledge that all that information about that person and what happened and the circumstances are there and the coroner can rely on really accurate information to make a finding. (Lauren)

Some participants, like Kate, appreciated that the coronial investigation triggered a more thorough investigation, although this process took time:

No friends gave statements until nearly a decade later… I must admit, [the investigating officer] was good, because it was done more accurately. Like all the statements were done. There were searches. There was none of that prior.

Protracted investigations created stress for some participants who retold their stories due to staff turnover and who required leave from work to attend lengthy inquests.

Conversely, some participants were “shocked” by how soon the inquest was held without adequate time to prepare emotionally. Two participants did not view timeframes of five years between disappearance and inquest as deleterious; both reported benefits from further searches conducted during this period, appropriate court updates, and court delays until family were available to attend the inquest. Elaine described the benefit of the inquest not being “rushed”:

[The investigating officer] recognised how strongly we felt that Josh was still alive and so he was on our side in that he didn’t rush through the inquest…That was all in our favour, in that they didn’t rush through the inquest in the hope that he’d turn up.

The issue of timely investigations was complex. For one participant who was opposed to the coronial investigation and finding of death, there would never be a right time for the inquest, while participants like Lauren felt that an inquest was necessary at some stage:

You have to draw a line in the sand at some stage I guess. Because, I mean, I wouldn’t want James to have disappeared for 50 years or 60 years and to have had no acknowledgement of his disappearance. I think at some stage you have to.

#### Best Practice – Timely Investigations

Some participants emphasised the importance of consulting families about the timing of inquests. A number of participants believed that negative family experiences of delays could be improved by more frequent communication about the reasons for delays. Families perceived a need to avoid delays that would result in a loss of crucial evidence. While some identified a need for more expedient investigations, others did not.

### A Public and Formal Court Environment

This theme comprised participants’ experiences of the public and formal court environment and had three sub-themes: (1) Facing the public: participants’ experiences of media and the public in court; (2) A formal environment: participants’ experiences of the court space; and (3) Best practice recommendations focused on the court environment.

#### Facing the Public

Some inquests did not attract media attention while others were highly publicised. The impact of media exposure was variable. In some cases, media coverage was seen as a valuable “tool” to invite information from the public. Some participants described distress in response to a lack of media coverage of the missing person: “Her face was never [known]–no one knows who she is” (Anne).

When journalists attended inquests, their behaviour was sometimes perceived as respectful, and sometimes “intrusive,” including instances where families were approached for comment in the court toilets. Two participants described media as “pushy,” and the “least helpful” aspect of the coronial process:

Me and [another family member] were sitting [in the court foyer] and realised – we were having a discussion and while we’re having a discussion the person that was sitting next to us all the time was a media person listening in and writing down what we were saying without even letting us know who she was. (Kate)

For others, a source of distress or concern, was media reports that were inaccurate, sensationalist, or that focused on details which removed the focus from the missing person. Kate expressed concern over media that might reduce the likelihood that community members would come forward with information: “The problem was putting [the person of interest in the newspaper]. Then people don’t go, ‘Okay, we need to ring Crime Stoppers’, because they already know who did it.”

Access to a private space at the court, away from media and the public, was “helpful” to a number of participants. One participant felt “comfortable” because they met with counsel assisting in a private room instead of the court foyer on the morning of the inquest, allowing the family to talk together. By contrast, two participants perceived it distressing when the person of interest was provided a private space at the court “for their protection” and “got to hide from the public” while the family lacked access to a similar space: “We had to walk past that gauntlet of media every morning, and sometimes at lunch time, and sometimes at the end of court. Every day we fronted it” (Jennifer).

For some participants it was the “court watchers” and “voyeurs” who were “disrespectful” and caused “distress,” in some instances pushing in front of family to get a seat. Hayley perceived the “least helpful” aspect of the process was having unknown people in the court taking notes:

All the back seats start to fill up… So we’re pouring our family love and everything [out] and opening our hearts and doing everything and there’s all these strangers up the back. …. I’m glad I didn’t notice it in the beginning… maybe I wouldn’t have spoken as freely.

One father felt it was “macabre” and added “strain” when an extended family member attended the inquest against the family’s express wishes creating a divisive potential.

#### A Formal Environment

Most described the courtroom as “formal” and many observed that people who are unprepared for this formality might feel “anxious” or “intimidated” or liken the environment to that of criminal courts seen on TV, thus misperceiving the purpose of the inquest to be about determining guilt. A number of participants expressed concern that this formality might inhibit family voice and the ability to contribute their views. However, some participants were able to “look past” this formality and “speak up” because they were used to public speaking; felt well-prepared about the court layout; or felt “at ease” due to “compassionate” treatment by court staff. Some participants described the “formality” of a courtroom as helpful to convey respect and the importance of the decisions made: “You feel like your missing loved one is being respected by the judiciary, by the law, as an individual” (Elaine).

#### Best Practice – The Court Environment

Most families recommended modernising the State Court space to make it more “comfortable” and “peaceful,” to improve amenities, and install private meeting spaces for families. Most of these measures were available in the new State Court at Lidcombe, thus participants’ comments on this topic were limited. Two participants appreciated the coroner’s flexibility in holding an inquest in their local court, which for them was an important component of best practice.

Views on the formality of the court were divided. Three participants felt the formality of a courtroom was best practice to convey an appropriate level of respect for the missing person, their family, and the decisions made:

If you are thrown in a little room or whatever, it just shows how much disrespect you have got for me, my family, and Josh… A courtroom is that. Don’t take me to a back room and sign a couple of papers and say: Sorry Mr. and Mrs. Matthews, I hope you don’t still feel the pain. (John)

By contrast, two participants suggested that where an inquest was non-suspicious, and where no witnesses other than family members were called, an option for non-public conferences to facilitate family input in the process would be helpful:

Maybe if there was some kind of criminal activity involved, yes, a courtroom. But it could well have been just done in a room with a table, like in a lawyer’s office or something. Informal setting might have been more—more congenial…It could be done in a much more relaxed atmosphere…because people would tend to talk more. (Marcus)

Most stated that education about the environment that included court visits, provision of pictures of the court, and explanations of the court layout, were “important” measures to ensure that families did not feel intimidated. Two participants recommended better management of the space to improve the court experience. Alex suggested a “priority seating” system:

There needs to be a formal attendance order for the court. I mean, obviously the legal counsel and police must get first priority, then I would say, the family second. Third, the media, and a long last and fourth, is the public.

### Coronial Outcomes

This theme focused on participants’ experiences of (1) Coronial findings, recommendations, and comments; (2) Experiences of events after the inquest; and (3) Best practice in relation to coronial outcomes.

#### Findings, Recommendations, and Comments

In some cases, coroners made formal comments in summation. For Anne, hearing the coroner formally acknowledge that “a lot of things went wrong with the [police] investigation” revealed the “truth” of her experience, and was the “most helpful” aspect of the coronial process. Lauren described the importance of hearing the coroner’s formal apology in regard to systemic failings:

The coroner said: every system has failed you…he was sort of shouldering the responsibility, saying that the system let you down, the police let you down, the hospital system let you down. So I thought that was a good thing and [my mother] thought that was acknowledgement… And he apologised. I think [my mother] recognised that as the state saying it.

The majority of participant comments centred on the impact of the coroner’s finding with respect to death, with few comments about findings on the circumstances of death. Of 12 participants who experienced a finding of death, responses varied. Some said the finding of death was “as expected” after receiving explanations about the likely findings prior to the inquest. Others described the finding of death as “devastating” even when anticipated. Three participants felt an open finding would have been “less distressing” and made more sense:

I mean that [an open finding] to me would not have been as shattering for us, in an emotional sense, as it was getting the advice that he was deceased. The deceased bit really has that ring of finality to it. Whereas the open finding, yeah well, that makes sense. Because no one knows what happened, so an open finding to us makes sense. (David)

For Maria a finding of death caused extreme distress and concern about how she would explain this to her daughter in future:

We live with this constant thing that is still alive… if she comes, she is going to say: Mum, why did you let him kill me?…Why didn’t you stop them writing a report of my death if I am not dead? That will be the first question she will ask me.

Some viewed the findings as an inevitable “rubber stamp” procedure, lacking in plausibility:

We’ve never from that day to this believed that. Who would? There’s always the chance that he could still be out there. So yes, so really it was just a matter of, I guess a formality that had to be done. (Marcus)

A number of participants described how a finding of death impacted members of the same family differently, each taking a different stance as to whether the finding provided “closure” or impacted their “hope” that the person was alive. Thus, coronial findings were perceived to have potential to exacerbate family tensions.

Of three participants whose inquest resulted in an open finding with respect to death, two described being “really pleased” and “comforted” because this allowed hope and further investigation: “The police still have him on their books … we are still looking for him” (John), and because they did not need a finding of death for “closure” or to manage estate. One participant expressed mixed feelings about the open finding, having decided before the inquest that any finding was “neither here nor there,” yet was “surprised” by the open finding, perceiving some benefit in the potential for continued police investigation and some detriment in “lingering paperwork” that could not be finalised.

One third of inquests resulted in recommendations; some related to requests for a reward or referrals to unsolved homicide. Some participants were distressed by a lack of expected recommendations, particularly in one case where the family wrote to the court prior to the inquest to request recommendations and was “upset” when no explanation was offered for rejecting their recommendations. Some were “happy” about recommendations for a reward or referrals to unsolved homicide, yet for some, these perceptions changed after the inquest.

#### After the Inquest

For some participants what happened after the inquest was the “most distressing.” Some described continuing confusion about their rights and a poor understanding of the conditions under which a further inquest might occur: “I still don’t know my rights as to what I can do here and whether I try for another inquest” (Anne).

A primary trigger for distress was a poor understanding of death certificate procedures after the inquest. All five participants who applied for a death certificate described this process as poorly explained and unnecessarily “traumatic” because they experienced lengthy delays, repeat applications for death certificates, and insensitive questions upon application, such as “he may not be dead. Have you tried searching for him?” (Lauren) Four participants decided against applying for a death certificate because they did not need a certificate to manage financial matters and because it represented a loss of hope of finding the person alive.

While some comfort arose from advice about the potential for future investigation:

It was made clear to us that [the finding of death] didn’t mean that the case was closed and that if any circumstances or facts ever came to light in the future which suggested he might be alive, that it would be pursued and investigated…. It gave [his mother] comfort to think they were not writing him off now…(Chloe)

Others described distress in response to unrealised expectations and a poor understanding of police investigations after a coronial finding. Some expected open findings, or referrals to unsolved homicide, to result in more immediate police investigation after an inquest, only to feel “distress” and “anger” when they learned of “delays” or “suspensions” in the police investigation pending new evidence. One felt “disappointed” when the reward was less than the coroner had recommended.

#### Best Practice – Outcomes

Many participants perceived it “vital” to be provided an opportunity to debrief with court professionals after the inquest. Some perceived pre and post inquest support and information as equally important components of best practice. Some suggested several points of contact with court professionals and/or counsellors after the inquest, to explain and discuss: (a) the findings, (b) implications for the ongoing police investigation, (c) issues that required clarification, (d) death certificate procedures, (e) the implementation of coronial recommendations, and (f) counselling referrals:

Perhaps after the process, maybe the following day: ‘How are you going?’ ‘Do you understand everything that came out of that?’ ‘Is there anything that happened that you don’t understand …?’ And the ‘Where to from here’…maybe one further contact after that, I suppose, focused on the practicalities… (Chloe)

## Discussion

This study explored family experiences of coronial procedures and, in line with TJ principles; investigated family perceptions of the impact of coronial proceedings on well-being and participant views of best practice. The findings can assist coroners who strive to prevent unnecessary trauma to impacted families (Dillon and Hadley, 2015). Overall, this research demonstrated variability in the format, experience and impact of inquests into missing people. Variability was evident in the timing, public exposure, level of information about the process, family input, access to support services, and coronial outcomes. The findings of the present study are consistent with research demonstrating that poor communication about the coronial process, a lack of preparation as to what to expect, preclusion of family voice, poor access to legal or counselling support, and insensitive treatment, are key contributors to family distress (Davis et al., 2002; Biddle, 2003; Victorian Parliament Law Reform Committee, 2006; Law Reform Commission of Western Australia, 2011; Ngo et al., 2018). However, this research further suggested that unnecessary distress can be reduced and in some cases meaningful, cathartic outcomes may be achieved if the following are offered to families: opportunities to be heard; treating them with compassion; and educating families about their rights, the coronial process and support services. While a number of studies drew overwhelmingly negative conclusions about poorly run inquests that amplified grief (Biddle, 2003), more recent studies described benefits to families who were included in inquests and provided access to vital information (Ngo et al., 2018; Spillane et al., 2019). This study revealed examples of participants who labelled aspects of their experience as “therapeutic” or “best practice” and others who perceived the process to compound their trauma. It is important to learn from all experiences, to examine “what works” as the court continues to evolve and to implement measures to assist families.

### The Importance of Being Heard, Education, and Humanity

A major outcome of this study was the perceived importance of providing opportunities for families to be heard through family statements, witness questioning, witness testimony, and the submission of suggestions for the investigation prior to the inquest. For some participants, feeling heard contributed to a meaningful experience and demonstrated the value of family members and their missing loved ones. These findings resonated with coronial research and texts that posit that families value opportunities to publicly state their personal views, and perceive justice and healing when provided opportunities to question witnesses or to express their views in court (Wertheimer, 2001; Freckelton and Ranson, 2006; Ngo et al., 2018). A significant body of procedural justice research demonstrated a similar link between positive therapeutic outcomes, judgments of fairness and procedures that allowed participant views to be “heard” (Lind and Tyler, 1988; Wemmers, 1996; Wemmers and Cyr, 2006). In the present study, the opportunity to be heard was perceived to be facilitated by: timely education, including explanations of family statements; access to evidence and support services; and sensitive treatment from court professionals. TJ scholars argue that legal outcomes are enhanced when courts attend to the well-being of participants and take steps to minimise harm (Casey and Rottman, 2000). Notably, hearing family views creates opportunity for families to contribute information that could aid coroners in their capacity as fact finders.

A number of families in this study described benefits arising from direct communication with court professionals who explained court proceedings and checked their understanding of the court process. Others described a detrimental impact when they were unclear about the process, their rights, and who to contact for advice. These findings correspond with research that described inadequate information and family liaison to exacerbate distress and that posited the benefit of comprehensive pre-inquest briefings, in addition to written information (Harwood et al., 2002; Spillane et al., 2019). Moreover, many participants in this study reported feeling better prepared for, and less anxious about, inquests, in cases where they were provided timely access to evidence in the brief and referrals to support services. These results accord with research specifying the benefit of timely referrals and allowing families “first hand” access to the evidence to enable full participation in proceedings, to minimise surprises, and to elucidate the scope of the investigation (Victorian Parliament Law Reform Committee, 2006; Matthews et al., 2016). The present study extended the coronial literature by describing particular aspects of support services that were helpful. For example, some participants derived value from: (a) minor legal assistance; (b) counsellors who were available to listen, to answer questions about the process, and to attend the inquest; and (c) events that provided opportunity for families to interact with court professionals and other families with experience and knowledge of the coronial process. These findings strengthened the argument for proactive contact from the court prior to an inquest to ensure families’ understanding of their rights and relevant contacts.

Many studies emphasised the importance of compassionate, kind treatment, to reduce harm and potentially transform the inquest experience into something meaningful (Davis et al., 2002; Snell and Tombs, 2011). While some research produced predominantly negative reflections about the distressing impact of “unsympathetic” coronial staff (Biddle, 2003, p. 1038; Snell and Tombs, 2011), the present study revealed largely positive reflections about the compassionate behaviour of court staff. Sensitive treatment was the factor most frequently cited as “most helpful” to well-being. These findings suggest that court personnel are potentially therapeutic agents and that “sensitive” behaviour and language has significant implications for family well-being. Displays of humanity were demonstrated through simple acts, including coroners who were patient, attentive, addressed people by name, and made statements to acknowledge the significance and emotional impact of the findings. These study outcomes further revealed the family view that the impact of legal terminology, including statements of legal “satisfaction,” could be improved through better preparation and explanation.

### Variable Perceptions of Timeliness and the Court Environment

Overwhelmingly the literature described extensive “delays” of months or years as a primary cause of family suffering, with researchers labelling delays of six years as “severe” (Ngo, 2016). No known research included views from families who “waited” for decades for an inquest, as was the case for a number of participants in the present study. Perceptions of timeliness of coronial investigations varied widely between family members, influenced by further collection of evidence, communication about the progress of the investigation, and family expectations and needs. Consistent with prior research, delays were perceived to exacerbate trauma when they contributed to a loss of crucial evidence and where families were uninformed of reasons for delays (Wertheimer, 2001; Davis et al., 2002; Harwood et al., 2002; Biddle, 2003; Victorian Parliament Law Reform Committee, 2006; Law Reform Commission of Western Australia, 2011). However, there were also families who perceived additional time helpful to conduct further investigations, to review the evidence, and to prepare emotionally for an inquest. While other studies conceptualise delays as traumatic in their potential to suspend grieving, the experience of suspended grief was described as typical, notwithstanding the coronial investigation. These findings suggested that distress could result from an inquest held too early or too late, and that for some, there would never be a right time. This finding poses a challenge for coroners, particularly in light of current expectations to finalise all coronial investigations within the national benchmark timeframe of 24 months, and where missing persons’ investigations are to be reported to coroners after 12 months in cases in which there are no signs of life (Federation of Community Legal Centres Victoria, 2013; Dartnall and Goodman-Delahunty, 2016). This finding highlighted the importance of appropriate communication when delays occur and the need for careful consideration of individual circumstances, balancing family views on the timing of proceedings with the impact of delays on the available evidence.

While many studies focus on the negative impact of media, public intrusion, and formal courtroom settings, families in the present study reported diverse responses to these factors. This research corroborated prior findings suggesting that family distress can result from the presence of unknown persons in court, inaccurate, or irrelevant media articles, and intrusive behaviours of journalists (Barraclough and Shepherd, 1977; Wertheimer, 2001; Davis et al., 2002; Biddle, 2003; Snell and Tombs, 2011; Spillane et al., 2019). However, this study also revealed cases in which the absence of public awareness of the missing person caused distress, and where media coverage was perceived as helpful to invite information from the public. Consistent with previous research, there were families in the current study who perceived a need for less formal settings to facilitate family input; and who observed the potential for courtrooms to trigger anxiety and unrealistic expectations that the process would produce determinations of guilt in cases where families were unprepared for the court environment (Biddle, 2003). Conversely, this research also revealed families who appreciated the formality of a respectful courtroom setting and who described the positive impact of compassionate treatment and appropriate education that assisted them to feel more comfortable in the court environment. Family suggestions for pre-inquest court visits, and explanations as to who might attend the inquest, echoed the recommendations of previous research (Law Reform Commission of Western Australia, 2011).

### Court Outcomes and Family Support After the Inquest

Family responses to coronial findings were similarly variable. Some participants perceived a finding of death as a mere “formality”, others perceived it as “traumatising” even when anticipated, and some welcomed open findings. These results suggested that responses to findings should neither be presumed nor underestimated. Ambiguous loss scholars suggested that an unworkable therapeutic approach requires families to hold on to only a single possibility–that the person is either here or gone (Boss, 2006, 2010). Forcing families toward one extreme is perceived as detrimental because this discounts the ongoing ambiguity and can lead to internalisation of blame by those who cannot accomplish an unreachable goal. Boss (2008, 2010) suggested that the key to resilience lies not in forcing acceptance of particular outcomes, but in approaches that: (a) demonstrate patience and validation of families’ variant views, (b) explore opportunities for meaning, (c) build capacity to hold conflicting ideas and feelings of ambiguity, and (d) allow families to share stories and to continue connections with the missing person. From an ambiguous loss perspective, forcing acceptance of coronial findings is unlikely to be congruent with emotional well-being. Rather, a non-judgmental approach that allows families to derive their own personal meaning from the inquest, share their views, and make sense of the findings in their own way and time, is more likely to minimise harm. Accordingly, this study demonstrated that some families appreciated non-judgmental approaches to the delivery of findings, including acknowledgement that the findings did not equate to closure.

Notably, some participants disputed or were dissatisfied with coronial findings. Nonetheless, some derived benefit from their views being heard, recognition of the missing person, a more thorough investigation, or a formal apology or comment that acknowledged systemic failings. These findings resonated with observations in other studies that it is not just the findings which impact families, but other attributes of the coronial process that are helpful or have utility (Roper, 2014). Families described distress arising from unrealised expectations that resulted from coronial outcomes and poorly explicated processes flowing from coronial findings. During the timeframe of this research, a plain language resource was developed to explain death certificate procedures in cases of missing people, addressing some of the concerns documented in this study (NSW Department of Justice Producer, 2017). These findings support the need for post-inquest debriefings that provide an opportunity to discuss court outcomes and to clarify the professionals responsible for providing families with information after an inquest.

### Limitations of the Study

This preliminary research provides rich descriptions of family experiences and a platform to identify future avenues for research. However, the study’s limitations must be acknowledged. First, the study sample was small and not representative of all families of missing people. Families should not draw conclusions about what will happen in their own inquest based on these findings, but should be encouraged to ask questions about these processes prior to an inquest.

Second, while a number of studies describe the prevalence of clinical diagnoses and symptoms in relatives of missing people, such as prolonged grief disorder, depression, PTSD, anxiety, and intrusive thoughts and images, the current study did not use validated psychometric measures to assess clinical well-being (Quirk and Casco, 1994; Campbell and Demi, 2000; Zvizdic and Butollo, 2001; Heeke et al., 2015; Lenferink et al., 2016). Impact on well-being was based on participants’ self-reports. Third, this study did not explore external factors that could impact family experiences of the coronial process, including prior experiences of the police investigation. Fourth, interviews were conducted at variable points in time after an inquest with some interviews conducted many years after an inquest, when participant memory and coronial processes may have changed.

A further limitation of this research stems from biases in the participant sample, all of whom spoke English, lived in Australia, and experienced an inquest in NSW. Families who live internationally or are non-English speaking, would likely face additional hurdles in navigating and comprehending this legal process. Families in other jurisdictions may experience different coronial procedures, for example, a coronial investigation into a missing person wherein findings are made “in chambers” (without an inquest).

### Implications for Research and Practice

An understanding of the psychological impact of suspected death proceedings could be enhanced by research that: (1) utilises clinical interviews and standardised psychometric measures to assess well-being before and after coronial proceedings, (2) explores family experiences of suspected death inquests versus inquests where a death is known, and (3) incorporates questions about participants’ perceptions of the police investigation. Future research that includes participants from other jurisdictions and backgrounds will further develop an understanding of the court process, best practice, and the utility of inquests in cases of missing people. Research that explores the views of court professionals will provide insight into best practice procedures that professionals perceive are feasible. This research illuminated variability in family responses and the importance of future research that explores different accounts of the same inquest, and the impact of inquests on non-family court participants whose well-being must also be weighed in the conduct of coronial proceedings.

The findings have several implications for coronial practice. Chief among these are the value of direct and comprehensive pre and post inquest briefings where feasible, to: (1) explain opportunities for families to express their views, (2) check family understanding of written notifications and court decisions, (3) identify the professionals responsible for relaying information to families, (4) allow opportunities for compassionate, “human” contact with the people in the courts, (5) familiarise families with the environment and the potentially distressing evidence that they may encounter, and (6) understand and address the individual needs and expectations of family members. Training that enhances professional understanding of ambiguous loss could support families to feel understood during proceedings. External jurisdictions may consider the merits of developing plain language resources that explain the unique features of coronial investigations and death certification procedures in cases of missing people, like those valued by participants in this study (Families and Friends of Missing Persons Unit, 2017; NSW Department of Justice Producer, 2017).

## Conclusion

This study uncovered a complex picture of family experiences, well-being, and inquests into missing people with four major outcomes. Firstly, no uniform picture of the experience of inquests emerged, nor of features of coronial investigations that are universally helpful or unhelpful. Secondly, the prevalent view was that the experience of the court system can be improved, and unnecessary distress mitigated, through: (a) timely and appropriate education, (b) opportunities for family input, (c) consultation with families, (d) compassionate treatment, and (e) referrals to support agencies. Thirdly, this study supported measures implemented by the courts to assist families, including family statements, opportunities to read the brief, and court-based counselling services. Finally, the study elucidated the distressing context of ambiguity and disenfranchised grief experienced by families of missing people, and the importance of coronial practice that provides clarity, reduces unnecessary ambiguity, and acknowledges and honours missing people.

## Data Availability Statement

Interview transcripts are not publicly available to preserve participant confidentiality. Transcript anonymization is infeasible due to the unique study population and public nature of inquests and police missing persons investigations. Contact the corresponding author for further information.

## Ethics Statement

This study involving human participants was reviewed and approved by the Charles Sturt University Human Research Ethics Committee (2015/274). Participants provided their written informed consent to participate in this study and for the publication of de-identified results in academic journals. In cases where de-identification was limited due to the public nature of the inquest, participants provided their written informed consent for the publication of any potentially identifiable data in this article.

## Author Contributions

SD designed the study, developed the research questions, recruited the participants, collected the data, led the analysis, and wrote the manuscript. JG-D was primary supervisor, contributed to the design and provided supervisory feedback throughout. JG was co-supervisor and compared transcripts with themes and the final report. JG-D and JG provided analytic feedback, and critically reviewed and edited the manuscript prior to submission.

## Disclaimer

All statements of fact, opinion, and analysis are those of the authors and do not reflect the official policy or position of the NSW Department of Communities and Justice.

## Conflict of Interest

SD was employed as a counsellor with FFMPU, NSW Department of Justice at the time this research was conducted.

The remaining authors declare that the research was conducted in the absence of any commercial or financial relationships that could be construed as a potential conflict of interest.

## Footnotes

1 https://www.missingpersons.justice.nsw.gov.au/

2 https://www.facebook.com/austmissingpersons/

3 https://www.facebook.com/Picnicformissing/

4 https://www.facebook.com/LeaveALightOninc/

5 http://www.coroners.justice.nsw.gov.au/Pages/support_services/info_families.aspx

## References

[B1] Abernethy J. Baker B. Dillon H. Roberts H. (2010). *Waller’s Coronial Law and Practice in New South Wales*, 4th Edn Sydney, NSW: LexisNexis Butterworths.

[B2] Bain W. (2011). *Inquest into the Death of Kelly Marie Fitzgerald.* Available at: https://coronialservices.justice.govt.nz/assets/Documents/Decisions/csu-2009-rot-000369-fitzgerald-kelly-marie.pdf (accessed June 21, 2019).

[B3] Barraclough B. M. Shepherd D. M. (1976). Public interest: private grief. *Br. J. Psychiatry* 129 109–113. 10.1192/bjp.129.2.109 963347

[B4] Barraclough B. M. Shepherd D. M. (1977). The immediate and enduring effects of the inquest on relatives of suicides. *Br. J. Psychiatry* 131 400–404. 10.1192/bjp.131.4.400 922267

[B5] Biddle L. (2003). Public hazards or private tragedies? An exploratory study of the effect of coroners’ procedures on those bereaved by suicide. *Soc. Sci. Med.* 56 1033–1045. 10.1016/S0277-9536(02)00097-7 12593876

[B6] Birgden A. Ward T. (2003). Pragmatic psychology through a therapeutic jurisprudence lens: psycholegal soft spots in the criminal justice system. *Psychol. Public Policy Law* 9 334–360. 10.1037/1076-8971.9.3-4.334

[B7] Boss P. (2002a). Ambiguous loss in families of the missing. *Lancet* 360(Suppl.), S39–S40. 10.1016/S0140-6736(02)11815-0 12504498

[B8] Boss P. (2002b). Ambiguous loss: working with families of the missing. *Fam. Process.* 41 14–17. 10.1111/j.1545-5300.2002.40102000014.x 11924081

[B9] Boss P. (2004). Ambiguous loss research, theory, and practice: reflections after 9/11. *J. Marriage Fam.* 66 551–566. 10.1111/j.0022-2445.2004.00037.x

[B10] Boss P. (2006). *Loss, Trauma, and Resilience: Therapeutic Work with Ambiguous Loss.* New York, NY: W.W. Norton.

[B11] Boss P. (2008). A tribute, not a memorial: understanding ambiguous loss. *SIGMOD Rec.* 37 19–20. 10.1145/1379387.1379392

[B12] Boss P. (2010). The trauma and complicated grief of ambiguous loss. *Pastoral Psychol.* 59 137–145. 10.1007/s11089-009-0264-0

[B13] Boss P. Carnes D. (2012). The myth of closure. *Fam. Process* 51 456–469. 10.1111/famp.12005 23230978

[B14] Braun V. Clarke V. (2006). Using thematic analysis in psychology. *Qual. Res. Psychol.* 3 77–101. 10.1191/1478088706qp063oa

[B15] Braun V. Clarke V. (2012). “Thematic analysis,” in *APA Handbook of Research Methods in Psychology*, Vol. 2 ed. Cooper H., (Washington, DC: American Psychological Association), 57–71.

[B16] Campbell C. L. Demi A. S. (2000). Adult children of fathers missing in action (MIA): an examination of emotional distress, grief, and family hardiness. *Fam. Relat.* 49 267–276. 10.1111/j.1741-3729.2000.00267.x

[B17] Casey P. Rottman D. B. (2000). Therapeutic jurisprudence in the courts. *Behav. Sci. Law* 18 445–457. 10.1002/1099-0798(2000)18:4<445::AID-BSL371>3.0.CO;2-J 11018778

[B18] Chapple A. Ziebland S. Hawton K. (2012). A proper, fitting explanation? Suicide bereavement and perceptions of the Coroner’s verdict. *Crisis* 33 230–238. 10.1027/0227-5910/a000139 22562861

[B19] Clark J. (2007). Adult siblings of long-term missing people: loss and “unending not knowing”. *Grief Matters Aust. J. Grief Bereave.* 10 16–19. Available at: https://research-repository.griffith.edu.au/handle/10072/18811 (accessed June 21, 2019). 3744954

[B20] Coronial Services of New Zealand (2016). *Declaring a Missing Person Legally Dead.* Available at: https://coronialservices.justice.govt.nz/about/declaring-a-missing-person-legally-dead/ (accessed June 21, 2019).

[B21] Dartnall S. Goodman-Delahunty J. (2016). The coronial investigation of suspected deaths: prevalence and outcomes in New South Wales. *J. Law Med.* 23 609–627. 27323638

[B22] Davis G. Lindsey R. Seabourne G. Griffiths-Baker J. (2002). *Experiencing Inquests. Home Office Research Study 241.* London: Home Office Research, Development and Statistics Directorate Available at: http://irep.ntu.ac.uk/id/eprint/27944 (accessed June 21, 2019).

[B23] Dillon H. (2015). *Raising Coronial Standards of Performance: Lessons from Canada, Germany & England.* Available at: https://www.churchilltrust. com.au/media/fellows/Dillon_H_2014_Best_practice_in_Australian_ coroners_courts.pdf (accessed June 21, 2019).

[B24] Dillon H. Hadley M. (2015). *The Australiasian Coroner’s Manual.* Sydney, NSW: The Federation Press.

[B25] Families and Friends of Missing Persons Unit (2017). *A Guide to Coronial Services in NSW for Families and Friends of Missing People.* Parramatta, NSW: NSW Department of Justice Available at: https://www.missingpersons.justice.nsw.gov.au/Documents/book_coroners-ol.pdf (accessed June 21, 2019).

[B26] Federation of Community Legal Centres Victoria (2013). *Saving Lives by Joining Up Justice: Why Australia needs Coronial Reform and How to Achieve it.* Melbourne, VIC: Federation of Community Legal Centres.

[B27] Freckelton I. (2006). Coronial law reform: the new wave. *J. Law Med.* 14 151–155. 17153519

[B28] Freckelton I. (2007). Death investigation, the coroner and therapeutic jurisprudence. *J. Law Med.* 15 242–253. 18035842

[B29] Freckelton I. (2016). Minimising the counter-therapeutic effects of coronial investigations: in search of balance. *QUT Law Rev.* 16 4–29. 10.5204/qutlr.v16i3.696

[B30] Freckelton I. Ranson D. (2006). *Death Investigation and the Coroner’s Inquest.* South Melbourne, VIC: Oxford University Press.

[B31] Glassock G. (2011). *Australian Families of Missing People: Narrating their Lived Experince.* Unpublished doctoral dissertation, University of New England, Armidale, NSW.

[B32] Hands T. (2012). Under the microscope: reforming Western Australia’s coronial system. *Brief* 39, 12–15. Available at: https://www.lrc.justice.wa.gov.au/_files/Hands%20T%20-%20Under%20the%20Microscope%20Brief%20April%202012.pdf (accessed June 21, 2019).

[B33] Harwood D. Hawton K. Hope T. Jacoby R. (2002). The grief experiences and needs of bereaved relatives and friends of older people dying through suicide: a descriptive and case-control study. *J. Affect. Disord.* 72 185–194. 10.1016/S0165-0327(01)00462-1 12200209

[B34] Heeke C. Stammel N. Knaevelsrud C. (2015). When hope and grief intersect: rates and risks of prolonged grief disorder among bereaved individuals and relatives of disappeared persons in Colombia. *J. Affect. Disord.* 173 59–64. 10.1016/j.jad.2014.10.038 25462397

[B35] Johnson R. Onwuegbuzie A. (2004). Mixed methods research: a research paradigm whose time has come. *Educ. Res.* 33 14–26. 10.3102/0013189X033007014

[B36] King M. S. (2008). Non-adversarial justice and the coroner’s court: a proposed therapeutic, restorative, problem-solving model. *J. Law Med.* 16 442–457. 19205307

[B37] Law Reform Commission of Western Australia (2011). *Review of Coronial Practice in Western Australia: Discussion Paper. Project no 100, 1–267.* Available at: http://www.lrc.justice.wa.gov.au/_files/P100-DP.pdf (accessed June 21, 2019).

[B38] Law Reform Commission of Western Australia (2012). *Review of coronial practice in Western Australia: Final report. Project no. 100, 1–176.* Available at: http://www.lrc.justice.wa.gov.au/_files/P100-FR.pdf (accessed June 21, 2019).

[B39] Legal Aid New South Wales (2016). *Statutory Review of the Coroners Act 2009: Draft proposals for legislative change. Submission to the NSW Department of Justice.* Available at: https://www.legalaid.nsw.gov.au/__data/assets/pdf_file/0004/25492/Legal-Aid-NSW-Submission-to-Coroners-Act-Statutory-Review-September-2016-.pdf (accessed October 7, 2019).

[B40] Lenferink L. Wessel I. de Keijser J. Boelen P. A. (2016). Cognitive behavioural therapy for psychopathology in relatives of missing persons: study protocol for a pilot randomised controlled trial. *Pilot Feasibility Stud.* 2 1–12. 10.1186/s40814-016-0055-1 27965839 PMC5153873

[B41] Lind E. A. Tyler T. R. (1988). *The Social Psychology of Procedural Justice.* New York, NY: Plenum Press.

[B42] Matthews L. Bohle P. Quinlan M. Kimber D. Ngo M. Finney Lamb C. (2017). *Death at Work: Improving Support for Families: Final Report.* Available at: http://sydney.edu.au/health-sciences/research/workplace-death/wds-report-june-2017.pdf (accessed June 21, 2019).

[B43] Matthews L. Fitzpatrick S. Quinlan M. Ngo M. Bohle P. (2016). Bereaved families and the coronial response to traumatic workplace fatalities: organizational perspectives. *Death Stud.* 40 191–200. 10.1080/07481187.2015.1115787 26681297

[B44] Matthews L. Quinlan M. Rawlings-Way O. Bohle P. (2012). The adequacy of institutional responses to death at work: experiences of surviving families. *Int. J. Disabil. Manag.* 6 37–48. 10.1375/jdmr.6.1.37

[B45] Ngo M. (2016). *Families’ Satisfaction with Information From the Coroners’ Courts About Fatal Work Incidents: How and Why did My Loved One Die?* Unpublished doctoral dissertation, University of Sydney, Sydney, NSW.

[B46] Ngo M. Matthews L. Quinlan M. Bohle P. (2018). Bereaved family members’ views of the value of coronial inquests into fatal work incidents. *OMEGA J. Death Dying* 1–12. 10.1177/0030222818819344 [Epub ahead of print]. 30572786

[B47] Nowell L. Norris J. White D. Moules N. (2017). Thematic anlaysis: striving to meet the trustworthiness criteria. *Int. J. Qual. Methods* 16 1–13. 10.1177/1609406917733847

[B48] NSW Department of Justice Producer (2017). *Applying for a Death Certificate for a Missing Person in NSW.* Available at: https://www.missingpersons.justice.nsw.gov.au/Documents/FS_Apply-for-death-cert.pdf (accessed June 21, 2019).

[B49] Parry A. Benger N. Weeramanthri T. (1996). Counselling services attached to Coroners’ offices across Australia. *Aborig. Isl. Health Work J.* 20 9–10.

[B50] Punch K. (1998). *Introduction to Social Research: Quantitative and Qualitative Approaches.* Thousand Oaks, CA: Sage Publications.

[B51] Quirk G. J. Casco L. (1994). Stress disorders of families of the disappeared: a controlled study in Honduras. *Soc. Sci. Med.* 39 1675–1679. 10.1016/0277-9536(94)90082-5 7846565

[B52] Robson C. McCartan K. (2016). *Real World Research*, 4th Edn Hokoben, New Jersey: Wiley.

[B53] Roper I. (2014). *Therapeutic Jurisprudence in the Coronial Jurisdiction.* Unpublished honours thesis, The Australian National University, Canberra, ACT.

[B54] Roper I. Holmes V. (2016). Therapeutic jurisprudence in the coronial jurisdiction. *J. Judic. Administration* 25 134–147. 24218777

[B55] Selby H. (1998). *The Inquest Handbook.* Leichhardt, NSW: The Federation Press.

[B56] Snell K. Tombs S. (2011). ‘How do you get your voice heard when no-one will let you?’ Victimisation at work. *Criminol. Crim. Justice* 11 207–223. 10.1177/1748895811401985

[B57] Spillane A. Matvienko-Sikar K. Larkin C. Corcoran P. Arensman E. (2019). How suicide-bereaved family members experience the inquest process: a qualitative study using thematic analysis. *Int. J. Qual. Stud. Health Well-Being* 14 1–10. 10.1080/17482631.2018.1563430 30693845 PMC6352946

[B58] Thornton P. (2016). *Guidance No. 18. Section 1(4) Reports: Investigation Without a Body.* Available at: https://www.judiciary.uk/wp-content/uploads/2013/09/guidance-no-18-investigation-without-body.pdf (accessed June 21, 2019).

[B59] UK Missing Persons Unit (n.d.). *Has Someone you Know Gone Missing?* [sic] *A Brief Guide to Coroners and Presumption of Death Procedures. Fact sheet 12.* Available at: https://missingpersons.police.uk/en-gb/resources/factsheets-for-families (accessed June 21, 2019).

[B60] Victorian Parliament Law Reform Committee (2006). *Coroners Act 1985: Final Report.* Available at: http://www.parliament.vic.gov.au/images/stories/committees/lawrefrom/coroners_act/final_report.pdf (accessed June 21, 2019).

[B61] Waller K. M. (1994). *Coronial Law and Practice in NSW*, 3rd Edn Sydney, NSW: Butterworths.

[B62] Wayland S. (2015). *‘I Still Hope, but What I Hope for Now has Changed’: A Narrative Inquiry Study of Hope and Ambiguous Loss When Someone is Missing.* Unpublished doctoral disseration, University of New England, Armidale, NSW.

[B63] Wemmers J. (1996). *Victims in the Criminal Justice System.* Amsterdam: Kugler.

[B64] Wemmers J. Cyr K. (2006). What fairness means to crime victims: a social psychological perspective on victim-offender mediation. *APCJ* 2 102–128.

[B65] Wertheimer A. (2001). *A Special Scar: The Experiences of People Bereaved by Suicide*, 2nd Edn Hove, East Sussex: Brunner-Routledge.

[B66] Zvizdic S. Butollo W. (2001). War-related loss of one’s father and persistent depressive reactions in early adolescents. *Eur. Psychol.* 6 204–214. 10.1027//1016-9040.6.3.204

